# Association of GLP-1 Receptor Agonist Exposure with Platelet Function in Patients with Diabetes and Peripheral Artery Disease After Lower-Extremity Revascularization

**DOI:** 10.3390/jcm15218591

**Published:** 2026-11-04

**Authors:** Radha Bansal, Shezan Fouzdar, Swechha Bhatt, Shiv Patel, Vanessa Sauer, Aseman Bagheri Sheshdeh, Prajakta Lokhande, Shaidah Deghan, Anahita Dua

**Affiliations:** Division of Vascular and Endovascular Surgery, Heart and Vascular Institute, Department of Surgery, Massachusetts General Hospital/Harvard Medical School, 55 Fruit Street, Boston, MA 02114, USA; rbansal3@mgh.harvard.edu (R.B.); sfouzdar@mgh.harvard.edu (S.F.); sbhatt12@mgh.harvard.edu (S.B.); spatel130@mgh.harvard.edu (S.P.); vsauer@mgh.harvard.edu (V.S.); abagherisheshdeh@mgh.harvard.edu (A.B.S.); plokhande@mgh.harvard.edu (P.L.); sdeghan@mgh.harvard.edu (S.D.)

**Keywords:** GLP-1 receptor agonists, peripheral artery disease, diabetes mellitus, lower-extremity revascularization, thromboelastography, platelet mapping, platelet reactivity

## Abstract

**Background/Objectives:** Glucagon-like peptide-1 receptor agonists (GLP-1RAs) improve cardiovascular outcomes in patients with type 2 diabetes and have emerging relevance in peripheral artery disease (PAD), but their association with platelet function after lower-extremity revascularization is uncertain. We evaluated whether active GLP-1RA exposure was associated with thromboelastography with platelet mapping (TEG-PM) and global hemostatic parameters in patients with diabetes and PAD. **Methods:** We retrospectively analyzed prospectively collected serial TEG data from adults with diabetes and PAD undergoing lower-extremity revascularization between December 2022 and December 2025. GLP-1RA exposure was defined as active use at each blood draw and treated as time-varying. Platelet outcomes were analyzed using linear mixed-effects models adjusted for nonlinear elapsed time, insulin, aspirin, clopidogrel, ticagrelor, age, sex, body mass index, renal function, coronary artery disease, and hypertension. False discovery rate (FDR) correction was applied within prespecified ADP and arachidonic acid (AA) outcome families. **Results:** The cohort included 178 patients contributing 462 scheduled measurements; 20 patients contributed 47 GLP-1RA-exposed measurements. At baseline, 13 of 169 patients were receiving a GLP-1RA. Adjusted GLP-1RA-associated differences were −1.98 percentage points for ADP aggregation (95% CI, −10.9 to 6.89; FDR-adjusted *p* = 0.815), −0.64 mm for ADP maximum amplitude (MA) (95% CI, −6.06 to 4.77; FDR-adjusted *p* = 0.815), −0.45 percentage points for AA aggregation (95% CI, −13.5 to 12.6; FDR-adjusted *p* = 0.947), and −1.63 mm for AA-MA (95% CI, −8.28 to 5.03; FDR-adjusted *p* = 0.947). Global TEG outcomes and GLP-1RA-by-insulin interactions were not significant. The strongest time interaction was observed for ADP-MA (omnibus *p* = 0.072; FDR-adjusted *p* = 0.145). **Conclusions:** No statistically significant associations were detected between active GLP-1RA exposure and pathway-specific platelet reactivity or global hemostatic parameters in this longitudinal PAD cohort. Estimates remained imprecise, and effects of potentially clinically relevant magnitude could not be excluded. The descriptive ADP-mediated temporal pattern warrants prospective evaluation in larger cohorts.

## 1. Introduction

Patients with diabetes undergoing lower-extremity revascularization for peripheral artery disease (PAD) represent a high-risk vascular population in whom thrombosis, inflammation, endothelial dysfunction, wound burden, and antiplatelet therapy converge. Antiplatelet therapy is central after revascularization, yet platelet responsiveness in PAD is heterogeneous and may be influenced by diabetes severity, renal dysfunction, procedural recovery, and concurrent medications [1,2]. Identifying medication exposures that may modify platelet function could therefore provide insight into thrombotic biology after revascularization.

GLP-1 receptor agonists are increasingly used in patients with type 2 diabetes, obesity, and cardiovascular disease. Their relevance to PAD has expanded after the STRIDE trial, in which semaglutide improved walking capacity in patients with symptomatic PAD and type 2 diabetes [3]. Beyond functional outcomes, recent PAD-specific studies and meta-analyses have reported lower risks of major adverse limb events, lower-extremity revascularization, amputation, and mortality among patients receiving GLP-1RAs, although much of this evidence remains observational [4]. These emerging clinical signals have increased interest in the mechanisms through which GLP-1RAs may influence vascular outcomes in PAD. However, the mechanisms underlying vascular benefit in PAD remain incompletely understood. Improvements in weight, glycemic control, endothelial function, and inflammation may contribute, while platelet function represents another biologically plausible pathway.

Experimental and translational studies support the biological effects of GLP-1 receptor signaling on platelets and thrombosis. GLP-1 receptor activation has been reported to inhibit platelet aggregation, reduce thrombus formation under flow conditions, and attenuate platelet activation through cyclic adenosine monophosphate/protein kinase A-dependent, nitric oxide-mediated, anti-inflammatory, and thromboxane-related pathways [5,6,7,8,9]. Emerging clinical evidence also supports the investigation of this pathway. In the randomized ELAID pilot study, liraglutide produced an early and transient attenuation of platelet aggregation responses to ADP, arachidonic acid, and collagen in patients with type 2 diabetes [10]. However, clinical evidence regarding the effects of GLP-1RAs on platelet function remains limited, and the clinical significance of these observations is not yet established [11]. Whether similar effects are detectable in patients with advanced PAD receiving antithrombotic therapy after lower-extremity revascularization is unknown.

TEG platelet mapping provides pathway-specific measures of adenosine diphosphate (ADP)- and arachidonic acid (AA)-mediated platelet response alongside global viscoelastic measures of clot initiation, clot strength, fibrin contribution, and fibrinolysis [12,13]. As a whole-blood assay, TEG-PM reflects platelet responsiveness within the broader hemostatic environment and may be influenced by assay variability, hematologic and coagulation factors, and concomitant antiplatelet therapy. These considerations are particularly relevant in patients with PAD after revascularization, in whom multiple clinical and pharmacologic factors may influence platelet function. We therefore evaluated whether active GLP-1RA exposure at the time of blood sampling was associated with differences in platelet-mapping and global TEG parameters among patients with diabetes and PAD undergoing lower-extremity revascularization.

## 2. Materials and Methods

### 2.1. Study Design and Population

We conducted a retrospective observational cohort study of prospectively collected data from patients with PAD and diabetes mellitus at Massachusetts General Hospital, Boston, Massachusetts, USA. Adults aged 60 years or older undergoing elective lower-extremity revascularization were prospectively enrolled between December 2022 and December 2025. All patients included in the present analysis had documented diabetes mellitus before or at the time of the index revascularization. The study was conducted in accordance with the Declaration of Helsinki and was approved by the institutional review board. Exclusion criteria included contraindication or allergy to antiplatelet therapy, inability to provide informed consent or undergo serial blood sampling, and pregnancy.

The analytic cohort included eligible patients with scheduled blood draws pre-operatively at baseline and post-operatively at 1 week, 1 month, 3 months, and 6 months. Because actual collection dates varied around nominal visits, elapsed days from the index revascularization were used for longitudinal modeling rather than the categorical visit alone.

### 2.2. GLP-1RA and Concomitant Medication Exposure

Medication exposure was ascertained from institutional health records at each TEG blood draw. GLP-1RA exposure was defined as documented active use at the time of the corresponding blood draw and was therefore treated as a time-varying exposure. Changes in exposure status during follow-up were identified by comparing active medication status across consecutive study measurements. Treatment duration, dose, and adherence were not incorporated into the present exposure definition. The timing of the most recent antiplatelet dose relative to blood collection was not systematically recorded or standardized.

GLP-1RAs represented in the analytic dataset included semaglutide, dulaglutide, liraglutide, and exenatide. Agents were analyzed as a class because individual-agent exposure counts were insufficient for stable drug-specific estimates. Concomitant medication exposures included insulin, aspirin, clopidogrel, ticagrelor, statin therapy, and anticoagulation. Anticoagulant exposure included apixaban, rivaroxaban, warfarin, dabigatran, and enoxaparin. Dual antiplatelet therapy (DAPT) was defined as concurrent aspirin plus either clopidogrel or ticagrelor. Because aspirin, clopidogrel, and ticagrelor were entered individually in the platelet-mapping models, DAPT was not included as an additional covariate. Changes in antithrombotic therapy during follow-up were therefore incorporated using the medication status documented at each corresponding blood draw.

### 2.3. Viscoelastic Blood Analysis

Whole-blood samples were collected at baseline before revascularization and during longitudinal follow-up at 1 week, 1 month, 3 months, and 6 months. Blood for global TEG assessment was collected into 2.7 mL sodium citrate vacutainer tubes, whereas samples for TEG platelet mapping (TEG-PM) were collected into 4.0 mL non-gel sodium heparin vacutainer tubes. In accordance with manufacturer guidelines, TEG-PM samples underwent a 30 min incubation period and were analyzed within 2 h of collection using the TEG 6s Hemostasis Analyzer (Haemonetics Corp., Boston, MA, USA). Global hemostatic outcomes in the present analysis were reaction time (R time), lysis at 30 min (LY30), citrated rapid TEG maximum amplitude (CRT-MA), and citrated functional fibrinogen maximum amplitude (CFF-MA). R time reflects the time to initial clot formation, CRT-MA reflects overall clot strength, CFF-MA reflects fibrin-mediated clot strength, and LY30 reflects fibrinolysis.

Platelet function was assessed using TEG platelet mapping (TEG-PM). ADP-mediated parameters reflect the P2Y12 pathway, whereas AA-mediated parameters reflect the cyclooxygenase/thromboxane pathway. Outcomes included ADP inhibition, ADP aggregation, ADP-MA, AA inhibition, AA aggregation, and AA-MA. Percent platelet inhibition was calculated as 100% minus percent platelet aggregation. Because inhibition and aggregation are mathematically complementary, aggregation was treated as the nonredundant inferential outcome, whereas inhibition was reported descriptively.

ADP- and AA-mediated inhibition/aggregation measures were not available for every blood draw because some platelet-mapping results were reported as inconclusive or uninterpretable. Because inhibition and aggregation are mathematically complementary, availability was identical for these two measures within each pathway. Agonist-specific MA measurements could remain available when inhibition/aggregation results were inconclusive.

### 2.4. Baseline Variables

Baseline demographic and clinical characteristics were obtained from the electronic medical record and prospectively maintained study records. Variables included age, sex, race/ethnicity, body mass index (BMI), tobacco use, hypertension, hyperlipidemia, coronary artery disease (CAD), history of myocardial infarction, renal status by estimated glomerular filtration rate category, functional impairment, current or prior cancer, prior deep vein thrombosis, prior stroke, prior pulmonary embolism, Rutherford category, intervention type, and baseline antithrombotic and metabolic medications. For longitudinal analyses, patient-level baseline covariates were carried across repeated measurements when subsequent records lacked the same baseline characteristic.

### 2.5. Statistical Analysis

Continuous baseline variables were summarized using means and standard deviations, and categorical variables using frequencies and percentages. Baseline characteristics were stratified by active GLP-1RA exposure at the baseline blood draw. Because the baseline exposed group was small, between-group baseline comparisons were interpreted descriptively.

The primary longitudinal platelet analyses used linear mixed-effects models with a random intercept for patient identifier. GLP-1RA exposure was modeled as a time-varying binary variable. Actual days from the index revascularization were modeled using a natural spline with internal knots at 30 and 90 days and boundary knots at 0 and 377 days. Adjusted models included concurrent insulin, aspirin, clopidogrel, and ticagrelor use, together with age, sex, BMI, renal-function category, CAD, and hypertension. Covariates were selected a priori based on their clinical relevance to GLP-1RA exposure, diabetes and PAD severity, and platelet function. Given the limited number of GLP-1RA-exposed patients, a sensitivity analysis was also performed using a more parsimonious model retaining actual postoperative time, modeled using the same spline specification as the primary analysis, together with concurrent insulin, aspirin, clopidogrel, and ticagrelor use. Models were estimated by maximum likelihood for model comparisons and by the prespecified mixed-effects framework for primary effect estimates. Missing TEG-PM measurements were not imputed; each longitudinal model included all available measurements for the corresponding outcome. Visit-level blood-draw availability was assessed separately from outcome-specific TEG-PM availability; platelet-mapping results reported as inconclusive or uninterpretable were considered unavailable only for the corresponding outcome.

For global TEG outcomes, random-intercept models for LY30 and CRT-MA produced boundary fits with an estimated between-patient variance of zero. To use one consistent inferential framework across global outcomes, all four global TEG outcomes were analyzed using linear models with CR2 patient-clustered robust variance estimation and Satterthwaite small-sample degrees of freedom. Adjusted global models included the same covariates as the platelet models, with additional adjustment for concurrent anticoagulant exposure. Anticoagulant exposure was included in the global TEG models because these parameters reflect coagulation-dependent whole-blood clot formation and strength and may be directly influenced by anticoagulant therapy. The primary platelet-mapping models focused on agonist-specific platelet responses and therefore adjusted for aspirin and P2Y12 inhibitors rather than anticoagulation. To assess whether exclusion of anticoagulant exposure influenced the platelet-mapping results, we performed an additional sensitivity analysis adding concurrent anticoagulant use to the primary platelet models.

Multiplicity was addressed using the Benjamini–Hochberg false discovery rate (FDR) procedure within prespecified biologically related, nonredundant outcome families. The ADP family included ADP aggregation and ADP-MA, and the AA family included AA aggregation and AA-MA. ADP and AA inhibition were reported descriptively with raw *p*-values because they are mathematically complementary to aggregation. The four global hemostatic outcomes formed a separate secondary FDR family.

Effect modification by concurrent insulin use was evaluated using GLP-1RA-by-insulin interaction terms for the four nonredundant platelet outcomes, with pathway-specific FDR correction of the interaction tests. Time-varying associations were explored using GLP-1RA-by-time spline interaction models. Omnibus likelihood-ratio tests compared models with and without the interaction terms. Estimated GLP-1RA contrasts at 0, 7, 30, 90, and 180 days were used only to describe the shape of the interaction and were not treated as independent inferential tests.

Analyses were performed in R version 4.5.2 (R Foundation for Statistical Computing, Vienna, Austria), including the lme4/lmerTest, emmeans, and clubSandwich packages.

## 3. Results

### 3.1. Cohort and Baseline Characteristics

The longitudinal cohort included 178 patients with diabetes and PAD, contributing 462 scheduled TEG measurements at baseline, 1 week, 1 month, 3 months, and 6 months. GLP-1RA exposure was active in 47 measurements from 20 patients. Baseline measurements were available for 169 patients, of whom 13 (7.7%) were receiving a GLP-1RA at the baseline blood draw. The remaining GLP-1RA-exposed patients contributed exposed measurements later in longitudinal follow-up, consistent with the time-varying exposure definition. Of the 20 patients who contributed at least one GLP-1RA-exposed measurement, 13 were exposed at all available study measurements and 7 contributed measurements during both exposed and unexposed periods. Among these 7 patients, 5 had at least one transition from unexposed to exposed status and 3 had at least one transition from exposed to unexposed status; one patient had transitions in both directions during follow-up.

Baseline characteristics are summarized in Table 1. Compared with patients not receiving a GLP-1RA at baseline, GLP-1RA users had a somewhat higher mean BMI (28.9 vs. 27.2 kg/m^2^), a higher proportion of women (46.2% vs. 30.1%), and more frequent insulin use (69.2% vs. 47.4%). All 13 baseline GLP-1RA users were classified as White, whereas race/ethnicity was more heterogeneous in the nonexposed group. Because only 13 patients were GLP-1RA-exposed at baseline, these differences were considered descriptive rather than evidence of statistically meaningful baseline imbalance.

Antithrombotic regimens varied during follow-up, with 76 of 178 patients contributing measurements under more than one antiplatelet regimen. These changes were incorporated into the longitudinal models using concurrent medication status at each blood draw; the visit-specific distribution of DAPT and anticoagulant use is shown in Table S1.

### 3.2. Primary Platelet-Mapping Analyses

Blood-draw availability varied across follow-up, with 169 measurements at baseline, 36 during the 1-week period, 118 at 1 month, 72 at 3 months, and 67 at 6 months. Missing follow-up blood draws primarily reflected nonattendance at scheduled research visits related to the burden and logistics of serial study assessments. Among blood draws that were obtained, additional outcome-specific missingness occurred when ADP- or AA-mediated inhibition/aggregation results were reported as inconclusive or uninterpretable. Because inhibition and aggregation are mathematically complementary, availability was identical for these measures within each pathway. Overall outcome-specific analytic sample sizes are summarized in Table S2, and visit-level blood-draw and outcome-specific TEG-PM availability are presented in Table S3.

In crude repeated-measures analyses, GLP-1RA exposure was associated with +4.85 percentage points of ADP inhibition (95% CI, −5.43 to 15.1; *p* = 0.354), −4.85 percentage points of ADP aggregation (95% CI, −15.1 to 5.43; *p* = 0.354), and −2.46 mm of ADP-MA (95% CI, −8.20 to 3.29; *p* = 0.400). AA estimates were +1.73 percentage points for inhibition (95% CI, −13.0 to 16.5; *p* = 0.818), −1.73 percentage points for aggregation (95% CI, −16.5 to 13.0; *p* = 0.818), and −3.56 mm for AA-MA (95% CI, −10.8 to 3.68; *p* = 0.334).

**Figure 1:**
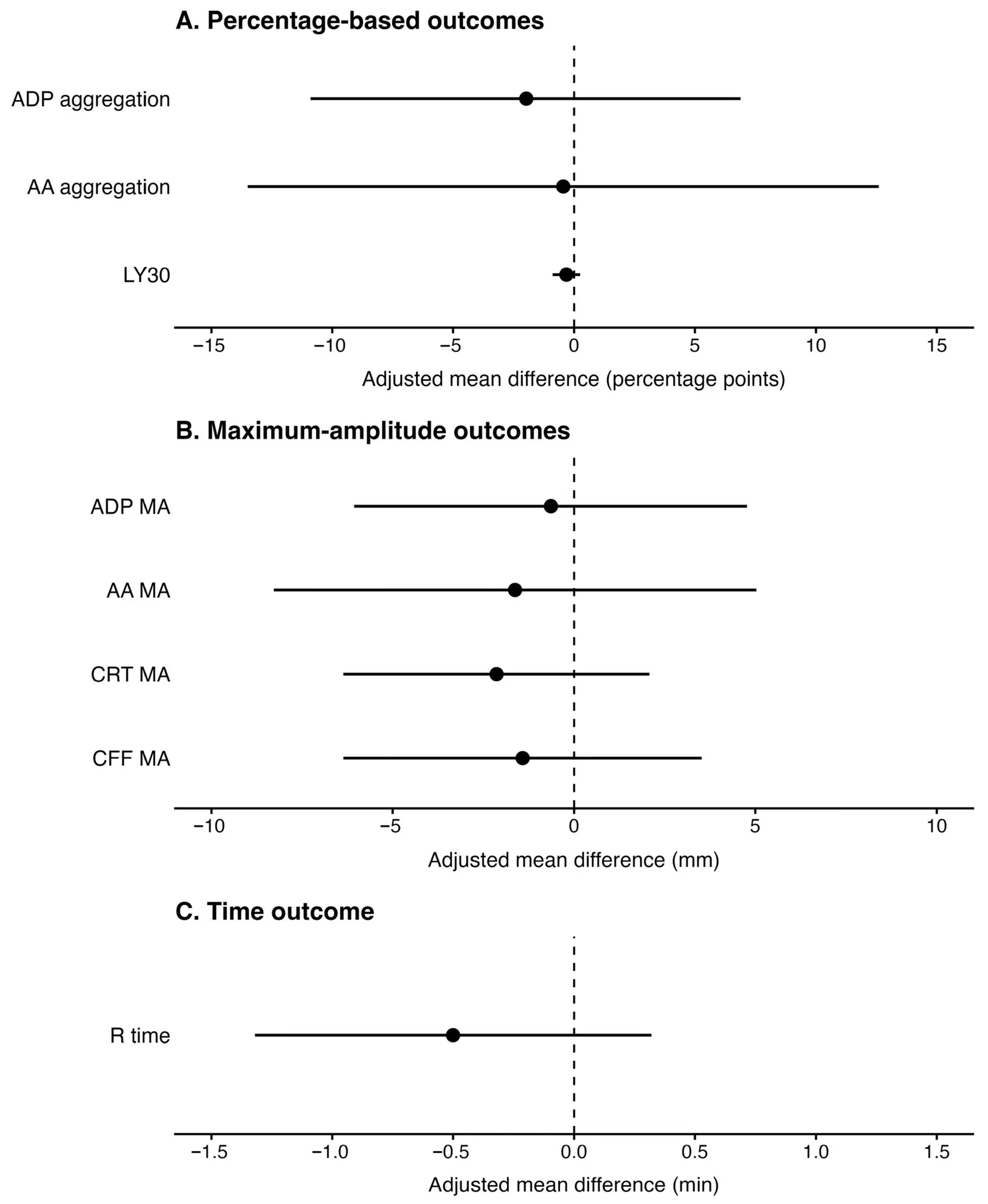
Adjusted associations between active GLP-1 receptor agonist exposure and nonredundant TEG outcomes. (**A**) Percentage-based outcomes. (**B**) Maximum-amplitude outcomes. (**C**) Time outcome.

After multivariable adjustment, GLP-1RA exposure was associated with +1.98 percentage points of ADP inhibition (95% CI, −6.89 to 10.9; *p* = 0.660), −1.98 percentage points of ADP aggregation (95% CI, −10.9 to 6.89; *p* = 0.660; FDR-adjusted *p* = 0.815), and −0.64 mm of ADP-MA (95% CI, −6.06 to 4.77; *p* = 0.815; FDR-adjusted *p* = 0.815). In the AA pathway, adjusted differences were +0.45 percentage points for inhibition (95% CI, −12.6 to 13.5; *p* = 0.947), −0.45 percentage points for aggregation (95% CI, −13.5 to 12.6; *p* = 0.947; FDR-adjusted *p* = 0.947), and −1.63 mm for AA-MA (95% CI, −8.28 to 5.03; *p* = 0.630; FDR-adjusted *p* = 0.947). Full crude and adjusted estimates are shown in Table 2 and Figure 1.

Points represent adjusted mean differences for GLP-1RA-exposed versus unexposed measurements; horizontal bars represent 95% confidence intervals. The dashed vertical line indicates no difference. Platelet outcomes were estimated using linear mixed-effects models; global outcomes used CR2 patient-clustered robust inference. ADP, adenosine diphosphate; AA, arachidonic acid; MA, maximum amplitude; TEG, thromboelastography; LY30, lysis at 30 min; CRT, citrated rapid TEG; CFF, citrated functional fibrinogen.

In a separate sensitivity analysis, additional adjustment for concurrent anticoagulant exposure did not materially alter the platelet-mapping estimates, and no associations were statistically significant after pathway-specific FDR correction (Table S4). In a parsimonious sensitivity analysis retaining postoperative time and concurrent insulin, aspirin, clopidogrel, and ticagrelor use—the variables most directly relevant to platelet reactivity—the findings were consistent with the primary analysis (Table S4).

### 3.3. Global Hemostasis

Global TEG outcomes showed no statistically significant associations with GLP-1RA exposure after multivariable adjustment and FDR correction. Estimates did not indicate a consistent shift in clot initiation, fibrinolysis, overall clot strength, or fibrin-mediated clot strength (Table 2).

### 3.4. Insulin Interaction Analysis

Of 47 GLP-1RA-exposed scheduled measurements, 33 occurred during concurrent insulin use. Formal interaction analyses did not demonstrate significant effect modification by concurrent insulin use after pathway-specific FDR correction (Table S5). Although ADP-mediated estimates were more negative during concurrent insulin use, the interaction tests did not support a differential association according to insulin status. Given the limited number of GLP-1RA-exposed patients, these analyses are considered exploratory.

### 3.5. Exploratory Time-Varying Associations

No GLP-1RA-by-time interaction reached statistical significance after pathway-specific FDR correction (Table S6). The strongest descriptive temporal pattern was observed for ADP-MA (omnibus *p* = 0.072; FDR-adjusted *p* = 0.145), with more negative estimates during later follow-up. GLP-1RA-exposed measurements remained limited across visits, including 2 during the 1-week period, 13 at 1 month, 11 at 3 months, and 8 at 6 months. Accordingly, the time-specific estimates are considered exploratory and are presented in Table S7.

## 4. Discussion

In this longitudinal cohort of patients with diabetes and PAD undergoing lower-extremity revascularization, we did not detect statistically significant associations between active GLP-1RA exposure and ADP- or AA-mediated platelet reactivity or with global TEG parameters after multivariable adjustment and pathway-specific multiplicity correction. Importantly, this study provides longitudinal estimates of platelet function during active GLP-1RA exposure in a high-risk PAD population in whom mechanistic data remain limited. Interpretation of the estimates is constrained by the small number of independently exposed patients: only 20 patients contributed 47 GLP-1RA-exposed measurements. Accordingly, the findings define the magnitude and uncertainty of the observed associations rather than demonstrating the absence of a platelet effect.

The ADP estimates were directionally compatible with modestly lower platelet reactivity during GLP-1RA exposure, although they remained imprecise. Prior studies in patients with PAD undergoing lower-extremity revascularization provide clinical context for the magnitude of this uncertainty. ADP aggregation was approximately 21 percentage points higher among patients who subsequently developed graft or stent thrombosis than among those without thrombosis, while ADP-MA was approximately 10 mm higher among patients who developed thrombotic or stenotic events [14,15]. In the present study, the 95% confidence intervals encompassed reductions of up to 10.9 percentage points in ADP aggregation and 6.06 mm in ADP-MA. Although these previously reported outcome-associated differences do not establish thresholds for clinically meaningful change, the width of the present confidence intervals indicates that platelet effects of potentially clinically relevant magnitude cannot be excluded.

The AA findings were particularly informative. Experimental work has suggested that GLP-1 receptor signaling may attenuate thromboxane-related platelet activation [7,8], providing a rationale for studying the AA pathway. However, the expanded multivariable analysis showed near-null adjusted differences for AA aggregation and a modest, imprecise reduction in AA-MA. This reinforces the need to distinguish mechanistic plausibility from evidence of an association in a heterogeneous clinical population.

The absence of a global TEG association was consistent across R time, LY30, CRT-MA, and CFF-MA. Prior clinical work in diabetes has suggested that GLP-1RA therapy may influence fibrinogen or selected viscoelastic measures [2], but our cohort differs substantially because of advanced PAD, revascularization, renal disease, tissue loss, inflammatory burden, and frequent antiplatelet and anticoagulant exposure. These competing influences may attenuate or obscure modest drug-related differences in whole-blood hemostasis.

The insulin interaction analysis did not support statistically significant effect modification. Although ADP aggregation and ADP-MA estimates were more negative among insulin-active observations than among observations without concurrent insulin, the formal interaction tests were not significant after FDR correction. This distinction is important because subgroup-specific point estimates can appear to differ even when evidence of true effect modification is weak, particularly when exposure is sparse.

In the time-interaction analysis, ADP-mediated outcomes showed a descriptive trajectory from higher platelet-mediated clot strength at baseline toward lower aggregation and ADP-MA during follow-up, with the largest estimated differences near 90 days. However, the omnibus ADP-MA interaction did not reach statistical significance (*p* = 0.072; FDR-adjusted *p* = 0.145), and the 90-day contrast remained imprecise. These findings should therefore be considered hypothesis-generating rather than evidence of a distinct 3-month treatment effect.

These results are clinically relevant because GLP-1RAs are increasingly used in patients with diabetes and established atherosclerotic disease, including PAD. The STRIDE trial demonstrated improved walking capacity with semaglutide in symptomatic PAD and type 2 diabetes [3], but the mechanisms responsible for those clinical benefits remain incompletely characterized. Our study does not demonstrate that platelet modulation is a major mechanism in patients after revascularization, but it provides longitudinal estimates of platelet function differences associated with active GLP-1 exposure and highlights the degree of uncertainty that future mechanistic studies must address. Because TEG-PM reflects laboratory measures of platelet responsiveness rather than clinical thrombotic or bleeding outcomes, the observed estimates cannot be assumed to translate into clinical benefit or harm and should not guide modification of antiplatelet therapy or other clinical management. Prospective studies with larger exposed cohorts, standardized assessment of GLP-1RA dose and duration, contemporaneous glycemic and inflammatory biomarkers, and clinical thrombotic and bleeding outcomes are needed.

### Strengths and Limitations

This study has several strengths, including serial prospectively collected TEG measurements, exposure ascertainment at the time of each blood draw, use of actual elapsed time rather than nominal visit alone, and a multiplicity strategy that did not treat inhibition and aggregation as independent outcomes. Several limitations remain. First, GLP-1RA exposure was observational and not randomized, so confounding by diabetes severity, obesity, renal function, prescribing patterns, medication access, and other clinical factors remains possible despite multivariable adjustment. Second, exposure is sparse: only 20 patients contributed 47 GLP-1RA-exposed scheduled measurements, and only 13 of 169 patients were exposed at baseline. Outcome-specific exposed counts ranged from 40 to 47 measurements, limiting precision, particularly for interaction analyses. Third, active medication use was determined from the medication record at each blood draw and does not establish adherence, dose, duration of therapy, or timing of the most recent dose. Fourth, GLP-1RAs were analyzed as a class because individual-agent counts were insufficient for stable drug-specific estimates; potential pharmacologic heterogeneity among semaglutide, dulaglutide, liraglutide, and exenatide could therefore not be evaluated. Fifth, longitudinal blood-draw availability decreased over time because some participants did not return for scheduled research follow-up visits, largely reflecting the burden of serial study assessments. Incomplete longitudinal follow-up may therefore have introduced selection bias related to follow-up availability. Among obtained blood draws, outcome availability also varied because some platelet-mapping results were inconclusive or uninterpretable; actual elapsed time was incorporated into the longitudinal models to account for variation around nominal study visits. Sixth, inhibition and aggregation are mathematically complementary and cannot be interpreted as independent corroborating outcomes; the multiplicity strategy explicitly treated aggregation and agonist-specific MA as nonredundant inferential outcomes. Seventh, the study did not directly measure thromboxane metabolites, platelet signaling markers, inflammatory biomarkers, endothelial function, or contemporaneous glycemic control. Finally, clinical thrombotic, bleeding, limb, and cardiovascular outcomes were not evaluated, so the findings should not be interpreted as evidence regarding the clinical efficacy or safety of GLP-1RA therapy.

## 5. Conclusions

This study provides longitudinal estimates of platelet-function and global hemostatic differences associated with active GLP-1RA exposure in patients with diabetes and PAD following lower-extremity revascularization. Among patients with diabetes and PAD following lower-extremity revascularization, active GLP-1RA exposure showed a directional pattern toward lower ADP-mediated platelet reactivity during follow-up, although no statistically significant associations were detected after multivariable adjustment and multiplicity correction. No consistent differences were observed in AA-mediated platelet reactivity or global hemostatic parameters, and neither concurrent insulin use nor postoperative time significantly modified the associations. The limited number of GLP-1RA-exposed patients resulted in imprecise estimates, with confidence intervals encompassing effects of potentially clinically relevant magnitude. Thus, the present findings do not exclude an antiplatelet effect of GLP-1RA therapy and provide estimates of its potential magnitude to inform larger prospective studies evaluating platelet biology and clinical outcomes in this high-risk population.

## Figures and Tables

**Table 1:** Baseline characteristics according to active GLP-1RA exposure at the baseline blood draw.

Characteristic	Overall (*N* = 169)	No GLP-1RA (*n* = 156)	GLP-1RA (*n* = 13)
Age, years, mean ± SD	69.0 ± 9.1	68.9 ± 9.3	70.6 ± 6.2
Female sex	53 (31.4%)	47 (30.1%)	6 (46.2%)
BMI, kg/m^2^, mean ± SD	27.3 ± 5.1	27.2 ± 5.1	28.9 ± 5.7
Race/ethnicity			
White	145 (85.8%)	132 (84.6%)	13 (100.0%)
Black or African American	12 (7.1%)	12 (7.7%)	0 (0.0%)
Asian	3 (1.8%)	3 (1.9%)	0 (0.0%)
Hispanic or Latino	4 (2.4%)	4 (2.6%)	0 (0.0%)
Other/unknown	5 (3.0%)	5 (3.2%)	0 (0.0%)
Tobacco use			
Never	41 (24.3%)	39 (25.0%)	2 (15.4%)
Former	97 (57.4%)	87 (55.8%)	10 (76.9%)
Current	31 (18.3%)	30 (19.2%)	1 (7.7%)
Hyperlipidemia	148 (87.6%)	135 (86.5%)	13 (100.0%)
Hypertension	157 (92.9%)	145 (92.9%)	12 (92.3%)
Coronary artery disease	106 (62.7%)	98 (62.8%)	8 (61.5%)
History of myocardial infarction	51 (30.2%)	46 (29.5%)	5 (38.5%)
Renal function			
eGFR > 90	67 (39.6%)	59 (37.8%)	8 (61.5%)
eGFR 60–89	41 (24.3%)	38 (24.4%)	3 (23.1%)
eGFR 30–59	42 (24.9%)	40 (25.6%)	2 (15.4%)
eGFR 15–29	5 (3.0%)	5 (3.2%)	0 (0.0%)
eGFR < 15 or dialysis	14 (8.3%)	14 (9.0%)	0 (0.0%)
Functional impairment	75/143 (52.4%)	72/132 (54.5%)	3/11 (27.3%)
Current cancer	10/143 (7.0%)	9/132 (6.8%)	1/11 (9.1%)
History of cancer	36 (21.3%)	32 (20.5%)	4 (30.8%)
History of DVT	24 (14.2%)	21 (13.5%)	3 (23.1%)
History of stroke	35 (20.7%)	32 (20.5%)	3 (23.1%)
History of pulmonary embolism	6 (3.6%)	5 (3.2%)	1 (7.7%)
Rutherford classification/clinical presentation			
Rutherford 0—Asymptomatic	2/158 (1.3%)	2/146 (1.4%)	0/12 (0.0%)
Rutherford 1–3—Claudication	41/158 (25.9%)	36/146 (24.7%)	5/12 (41.7%)
Rutherford 4–6—Chronic limb-threatening ischemia	115/158 (72.8%)	108/146 (74.0%)	7/12 (58.3%)
Intervention type			
Endovascular	116 (68.6%)	108 (69.2%)	8 (61.5%)
Open	34 (20.1%)	31 (19.9%)	3 (23.1%)
Hybrid	19 (11.2%)	17 (10.9%)	2 (15.4%)
Insulin	83 (49.1%)	74 (47.4%)	9 (69.2%)
Aspirin	130 (76.9%)	119 (76.3%)	11 (84.6%)
Clopidogrel	61 (36.1%)	58 (37.2%)	3 (23.1%)
Ticagrelor	3 (1.8%)	3 (1.9%)	0 (0.0%)
Statin	140 (82.8%)	128 (82.1%)	12 (92.3%)
Anticoagulation	33 (19.5%)	29 (18.6%)	4 (30.8%)

Values are *n* (%) unless otherwise indicated. For variables with missing data, values are presented as *n*/*N* (%), where *N* represents the number of patients with available data for that characteristic. GLP-1RA, glucagon-like peptide-1 receptor agonist; BMI, body mass index; eGFR, estimated glomerular filtration rate; and DVT, deep vein thrombosis. GLP-1RA exposure in Table 1 reflects active use at the baseline blood draw; exposure was time-varying in longitudinal analyses, in which 20 patients contributed 47 GLP-1RA-exposed measurements. Rutherford classification/clinical presentation was available for 158 patients and was categorized as Rutherford 0 (asymptomatic), Rutherford 1–3 (claudication), and Rutherford 4–6 (chronic limb-threatening ischemia).

**Table 2:** Unadjusted and adjusted associations of active GLP-1RA exposure with platelet-mapping and global TEG outcomes.

Outcome	*N* (Patients)	Exposed *N* (Patients)	Unadjusted Estimate (95% CI)	Unadjusted *p*-Value	Adjusted Estimate (95% CI)	Adjusted *p*-Value/FDR-Adjusted *p*-Value
ADP inhibition, %	340 (164)	40 (20)	+4.85 (−5.43, 15.1)	0.354	+1.98 (−6.89, 10.9	0.660/—
ADP aggregation, %	340 (164)	40 (20)	−4.85 (−15.1, 5.43)	0.354	−1.98 (−10.9, 6.89)	0.660/0.815
ADP-MA, mm	448 (176)	46 (20)	−2.46 (−8.20, 3.29)	0.400	−0.64 (−6.06, 4.77)	0.815/0.815
AA inhibition, %	343 (164)	40 (20)	+1.73 (−13.0, 16.5)	0.818	+0.45 (−12.6, 13.5)	0.947/—
AA aggregation, %	343 (164)	40 (20)	−1.73 (−16.5, 13.0)	0.818	−0.45 (−13.5, 12.6)	0.947/0.947
AA-MA, mm	451 (175)	47 (20)	−3.56 (−10.8, 3.68)	0.334	−1.63 (−8.28, 5.03)	0.630/0.947
R time, min	413 (171)	42 (19)	−0.23 (−1.14, 0.68)	0.597	−0.50 (−1.32, 0.32)	0.217/0.405
LY30, %	420 (173)	40 (19)	−0.46 (−1.03, 0.12)	0.111	−0.32 (−0.89, 0.25)	0.255/0.405
CRT-MA, mm	427 (175)	43 (20)	−2.96 (−7.03, 1.11)	0.144	−2.14 (−6.36, 2.08)	0.304/0.405
CFF-MA, mm	412 (169)	43 (20)	−1.71 (−6.57, 3.16)	0.472	−1.42 (−6.36, 3.52)	0.558/0.558

Estimates represent GLP-1RA minus no GLP-1RA. Adjusted models were linear mixed-effects models with a patient-specific random intercept and adjustment for nonlinear elapsed time, insulin, aspirin, clopidogrel, ticagrelor, age, sex, BMI, renal-function category, CAD, and hypertension. Global outcomes used CR2 patient-clustered robust inference and additionally adjusted for anticoagulation. FDR correction was applied within the ADP, AA, and global-hemostasis families; inhibition outcomes are shown with raw *p*-values only because inhibition is mathematically complementary to aggregation. ADP, adenosine diphosphate; AA, arachidonic acid; MA, maximum amplitude; TEG, thromboelastography; LY30, lysis at 30 min; CRT, citrated rapid TEG; CFF, citrated functional fibrinogen; CI, confidence interval; and FDR, false discovery rate.

## Data Availability

The patient-level data are not publicly available because they contain protected health information and are subject to institutional and IRB restrictions. Requests regarding de-identified data may be directed to the corresponding author and will be considered in accordance with institutional policies and applicable approvals.

## Supplementary Materials

The following supporting information can be downloaded at: https://www.mdpi.com/article/10.3390/jcm15218591/s1, Table S1, antithrombotic therapy across longitudinal study measurements; Table S2, outcome-specific analytic sample sizes; Table S3, Visit-level blood-draw and TEG-PM outcome availability; Table S4, sensitivity analyses of the association between active GLP-1RA exposure and platelet-mapping outcomes; Table S5, GLP-1RA-by-insulin interaction analysis for nonredundant platelet outcomes; Table S6, exploratory GLP-1RA-by-time interaction analyses; Table S7, descriptive GLP-1RA contrasts at prespecified postoperative times.
