# Supplementary material for: Association of GLP-1 Receptor Agonist Exposure with Platelet Function in Patients with Diabetes and Peripheral Artery Disease After Lower-Extremity Revascularization
Source: J Clin Med. 2026 Nov 4;15(21):8591. doi: 10.3390/jcm15218591 (PMC13764424; doi:10.3390/jcm15218591)
Supplement: Supplementary file 1 [file jcm-15-08591-s001.zip › jcm-4611734-supplementary.pdf]

Supplementary Tables

Table S1. Antithrombotic therapy across longitudinal study measurements

Visit	Measurements, n	Aspirin only, n (%)	DAPT, n (%)	P2Y12 inhibitor only, n (%)	Neither, n (%)	Anticoagulation, n (%)
Baseline	169	85 (50.3)	45 (26.6)	19 (11.2)	20 (11.8)	33 (19.5)
1 week	36	13 (36.1)	13 (36.1)	6 (16.7)	4 (11.1)	15 (41.7)
1 month	118	36 (30.5)	56 (47.5)	22 (18.6)	4 (3.4)	60 (50.8)
3 months	72	11 (15.3)	44 (61.1)	14 (19.4)	3 (4.2)	36 (50.0)
6 months	67	20 (29.9)	33 (49.3)	12 (17.9)	2 (3.0)	37 (55.2)

Antithrombotic exposure reflects documented active therapy at the corresponding blood draw. DAPT was defined as concurrent aspirin plus either clopidogrel or ticagrelor. P2Y12 inhibitor only indicates clopidogrel or ticagrelor without concurrent aspirin. Medication exposures were treated as time-varying in the longitudinal analyses. Overall, 76 of 178 patients had more than one antiplatelet regimen across their available study measurements. Anticoagulation included apixaban, rivaroxaban, warfarin, dabigatran, or enoxaparin. DAPT, dual antiplatelet therapy.

Table S2. Outcome-specific analytic sample sizes.

Outcome	Observations	Patients	GLP-1RA observations	GLP-1RA patients
ADP inhibition	340	164	40	20
ADP aggregation	340	164	40	20
ADP-MA	448	176	46	20
AA inhibition	343	164	40	20
AA aggregation	343	164	40	20
AA-MA	451	175	47	20
R time	413	171	42	19
LY30	420	173	40	19
CRT-MA	427	175	43	20
CFF-MA	412	169	43	20

GLP-1RA, glucagon-like peptide-1 receptor agonist; ADP, adenosine diphosphate; AA, arachidonic acid; MA, maximum amplitude; LY30, lysis at 30 min; CRT, citrated rapid thromboelastography; CFF, citrated functional fibrinogen.

Table S3. Visit-level blood-draw and TEG-PM outcome availability

Visit	Blood draws obtained, n	ADP aggregation, n (%)	ADP-MA, n (%)	AA aggregation, n (%)	AA-MA, n (%)
Baseline	169	127 (75.1)	162 (95.9)	127 (75.1)	161 (95.3)
1 week	36	22 (61.1)	36 (100.0)	22 (61.1)	36 (100.0)
1 month	118	78 (66.1)	115 (97.5)	79 (66.9)	117 (99.2)
3 months	72	55 (76.4)	69 (95.8)	57 (79.2)	71 (98.6)
6 months	67	58 (86.6)	66 (98.5)	58 (86.6)	66 (98.5)

Percentages are calculated from the scheduled blood draws available at each visit. ADP- and AA-mediated inhibition/aggregation measures were unavailable for some blood draws when the corresponding platelet-mapping result was reported as inconclusive or uninterpretable. Because inhibition and aggregation are mathematically complementary, availability was identical within each pathway. Missing TEG-PM values were not imputed; each outcome-specific longitudinal model used all available measurements for that parameter. Missing follow-up blood draws primarily reflected nonattendance at scheduled research visits, whereas lower outcome-specific availability among obtained blood draws reflected inconclusive or uninterpretable platelet-mapping results. ADP, adenosine diphosphate; AA, arachidonic acid; MA, maximum amplitude.

Table S4. Sensitivity analyses of the association between active GLP-1RA exposure and platelet-mapping outcomes

Outcome	Primary adjusted estimate (95% CI)	P / FDR-adjusted P	Parsimonious estimate (95% CI)	P / FDR-adjusted P	Anticoagulant-adjusted estimate (95% CI)	P / FDR-adjusted P
ADP aggregation, %	-1.98 (-10.9, 6.89)	.660 / .815	-2.18 (-11.6, 7.28)	.650 / .729	-1.57 (-10.4, 7.27)	.726 / .899
ADP-MA, mm	-0.64 (-6.06, 4.77)	.815 / .815	-0.99 (-6.63, 4.64)	.729 / .729	-0.35 (-5.76, 5.06)	.899 / .899
AA aggregation, %	-0.45 (-13.5, 12.6)	.947 / .947	+1.81 (-11.4, 15.0)	.787 / .787	-0.56 (-13.7, 12.5)	.932 / .932
AA-MA, mm	-1.63 (-8.28, 5.03)	.630 / .947	-1.31 (-8.01, 5.39)	.701 / .787	-1.55 (-8.21, 5.11)	.648 / .932

Estimates represent the adjusted difference for GLP-1RA-exposed versus unexposed measurements. Primary models adjusted for actual postoperative time using a natural spline, concurrent insulin, aspirin, clopidogrel, and ticagrelor use, age, sex, body mass index, renal-function category, coronary artery disease, and hypertension. Parsimonious sensitivity models retained postoperative time using the same spline specification and concurrent insulin, aspirin, clopidogrel, and ticagrelor use. Anticoagulant-adjusted sensitivity models used the primary model specification with additional adjustment for concurrent anticoagulant exposure. Benjamini-Hochberg FDR correction was applied separately within the ADP and AA outcome families. All sensitivity models were nonsingular. GLP-1RA, glucagon-like

peptide-1 receptor agonist; ADP, adenosine diphosphate; AA, arachidonic acid; MA, maximum amplitude; CI, confidence interval; FDR, false discovery rate.

Table S5. GLP-1RA-by-insulin interaction analysis for nonredundant platelet outcomes.

Outcome	No insulin: GLP-1RA difference (95% CI)	P	Insulin active: GLP-1RA difference (95% CI)	P	Interaction estimate (95% CI)	Interaction P / FDR P
ADP aggregation, %	+5.76 (-9.44, 20.96)	.456	-5.71 (-16.66, 5.24)	.306	-11.5 (-28.5, 5.59)	.187 / .195
ADP-MA, mm	+4.20 (-5.24, 13.64)	.382	-2.74 (-9.24, 3.76)	.408	-6.94 (-17.4, 3.56)	.195 / .195
AA aggregation, %	+2.72 (-19.3, 24.7)	.808	-1.96 (-17.9, 14.0)	.809	-4.68 (-29.1, 19.7)	.706 / .859
AA-MA, mm	-0.84 (-12.21, 10.54)	.885	-1.96 (-9.81, 5.89)	.624	-1.12 (-13.5, 11.3)	.859 / .859

Stratum-specific contrasts are GLP-1RA minus no GLP-1RA from models containing the GLP-1RA-by-insulin interaction. The interaction term is the inferential test of effect modification. FDR correction was applied separately within ADP and AA pathways.

Table S6. Exploratory GLP-1RA-by-time interaction analyses.

Outcome	Observations	Patients	Omnibus χ^2	Interaction P	FDR P
ADP aggregation	340	164	4.43	.219	.219
ADP-MA	448	176	6.99	.072	.145
AA aggregation	343	164	1.41	.702	.702
AA-MA	451	175	3.73	.293	.585

Time was modeled continuously using a natural spline. Omnibus likelihood-ratio tests compared models with versus without GLP-1RA-by-time interaction terms. FDR correction was applied separately within ADP and AA pathways.

Table S7. Descriptive GLP-1RA contrasts at prespecified postoperative times.

Outcome	Day	Estimate	95% CI	Descriptive P
ADP aggregation, %	0	+8.07	-5.56 to 21.70	0.245
ADP aggregation, %	7	+5.27	−6.52 to 17.10	.380
ADP aggregation, %	30	-4.46	-18.60 to 9.67	0.535
ADP aggregation, %	90	-11.00	-24.10 to 2.06	0.099
ADP aggregation, %	180	-8.39	-24.70 to 7.87	0.311
ADP-MA, mm	0	+6.60	-1.57 to 14.80	0.113
ADP-MA, mm	7	+4.75	−2.33 to 11.80	0.188
ADP-MA, mm	30	-1.79	-10.00 to 6.43	0.669
ADP-MA, mm	90	-7.31	-15.00 to 0.39	0.063
ADP-MA, mm	180	-5.66	-15.30 to 3.97	0.249

Outcome	Day	Estimate	95% CI	Descriptive P
AA aggregation, %	0	+6.57	-12.80 to 26.00	0.506
AA aggregation, %	7	+3.64	−13.40 to 20.70	0.674
AA aggregation, %	30	-5.88	-25.90 to 14.20	0.565
AA aggregation, %	90	-4.57	-23.20 to 14.00	0.630
AA aggregation, %	180	-0.42	-23.20 to 22.30	0.971
AA-MA, mm	0	+4.26	-5.37 to 13.90	0.385
AA-MA, mm	7	+1.93	−6.56 to 10.40	0.655
AA-MA, mm	30	-5.70	-15.30 to 3.92	0.245
AA-MA, mm	90	-5.15	-14.10 to 3.86	0.262
AA-MA, mm	180	-1.72	-12.70 to 9.31	0.760

Time-specific contrasts are descriptive estimates used to characterize the interaction shape; inference is based on the omnibus interaction tests in Table S6. Estimates represent GLP-1RA minus no GLP-1RA.
